# Inflammatory Responses Reprogram T_REGS_ Through Impairment of Neuropilin-1

**DOI:** 10.1038/s41598-019-46934-x

**Published:** 2019-07-18

**Authors:** Tim Hung-Po Chen, Manoj Arra, Gabriel Mbalaviele, Gaurav Swarnkar, Yousef Abu-Amer

**Affiliations:** 1 Department of Orthopaedic Surgery, Washington University School of Medicine, St. Louis, MO 63110 USA; 2 Cell Biology & Physiology, Washington University School of Medicine, St. Louis, MO 63110 USA; 3 Shriners Hospital for Children, St. Louis, MO 63110 USA; 4 Bone and Mineral Division, Department of Medicine, Washington University School of Medicine, St. Louis, MO 63110 USA

**Keywords:** Cell signalling, Osteoimmunology

## Abstract

Chronic inflammatory insults compromise immune cell responses and ultimately contribute to pathologic outcomes. Clinically, it has been suggested that bone debris and implant particles, such as polymethylmethacrylate (PMMA), which are persistently released following implant surgery evoke heightened immune, inflammatory, and osteolytic responses that contribute to implant failure. However, the precise mechanism underlying this pathologic response remains vague. T_REGS_, the chief immune-suppressive cells, express the transcription factor Foxp3 and are potent inhibitors of osteoclasts. Using an intra-tibial injection model, we show that PMMA particles abrogate the osteoclast suppressive function of T_REGS_. Mechanistically, PMMA particles induce T_REG_ instability evident by reduced expression of Foxp3. Importantly, intra-tibial injection of PMMA initiates an acute innate immune and inflammatory response, yet the negative impact on T_REGS_ by PMMA remains persistent. We further show that PMMA enhance T_H_17 response at the expense of other T effector cells (T_EFF_), particularly T_H_1. At the molecular level, gene expression analysis showed that PMMA particles negatively regulate Nrp-1/Foxo3a axis to induce T_REG_ instability, to dampen T_REG_ activity and to promote phenotypic switch of T_REGS_ to T_H_17 cells. Taken together, inflammatory cues and danger signals, such as bone and implant particles exacerbate inflammatory osteolysis in part through reprogramming T_REGS_.

## Introduction

Inflammatory osteolysis is a major complication of orthopedic joint implants^1^. Debris released from these implants trigger immune and inflammatory responses that promote recruitment of macrophages and osteoclasts to the injury site^2^. This cell- and cytokine-based pathologic response accelerates bone erosion around the implant leading to loosening and ultimately failure of implants, which poses high morbidity and mortality risks. Despite extensive efforts, the details of the biologic response to implant debris remain enigmatic and the complete repertoire by which orthopedic particles modulate cell lineages to enhance inflammation and exacerbate osteolysis remains poorly understood.

The cellular response to inflammatory triggers, including PMMA and bone particles, entails recruitment and activation of myeloid and immune cells such as macrophages, dendritic cells, granulocytes and lymphocytes. The ensuing inflammatory response is consistent with release of wear particles from implants and intensifies with increased particle burden akin to chronic response^3–5^. These particles are largely associated with inflammatory macrophages and osteoclasts at the implant-bone interface. However, the presence of multitude of other immune cell types, especially lymphocytes was also noted^6–10^. T lymphocytes mediate the adaptive phase of the immune response and are typically activated by antigen presenting cells such as dendritic cells. The most frequent subsets of these cells in inflammatory loci include T regulatory (T_REG_) and T helper (T_H_) cells. T_REG_ cells express the transcription factor Foxp3 and elicit inhibitory activity. On the other hand, T_H_ cells can differentiate based on their response to specific sets of factors in their microenvironment into T_H_1, T_H_17, or T_H_2 subtypes. Whereas T_H_1 and T_H_17 respectively secrete TNFα, IFNγ and IL-17A among many other pro-inflammatory cytokines, T_H_2 cells secrete primarily anti-inflammatory mediators including IL-4 and IL-10^11^.

The specific contribution of lymphocytes to wear debris-induced inflammatory osteolysis remains controversial. In this regard, circumstantial findings point to potential direct lymphocyte involvement as well as indirect action through secretion of pro-inflammatory and pro-osteoclastogenic factors such as RANKL, IL-17A, M-CSF or anti-osteoclastogenic factors such as IFNγ and IL-4^9,12–15^. Notably, we have shown recently that mice harboring loss-of-function mutant foxp3 in T cells display severe osteopenia^16^. This finding led us to speculate that reduced immunosuppression is conceivably one of the major reprogramming of immune cells elicited by PMMA particles during the progression of inflammatory osteolysis. Hence, the goal of this study is to decipher the pathologic mechanisms by which implant debris modulate lymphocytes to exacerbate inflammatory osteolysis. To this end, we show that PMMA particles, likely through a stress mechanism, modulate T cell activation by down-regulating Foxp3 function, resulting with T cell phenotypic switch from T_REG_ into pathogenic T_H_ cells with enhanced NF-κB activity. Mechanistically, we provide evidence that particles down regulate expression of Nrp-1 leading to reprogramming of T_REGS_. This change not only renders T_REGS_ incapable of inhibiting osteoclasts, but they secrete pro-osteoclastogenic and pro-inflammatory factors that expand the myeloid-progenitor population and enhance osteoclastogenesis.

## Results

### PMMA particles induce NF-κB activity and alter bone marrow cellularity toward reduced immunosuppression

**Figure 1:**
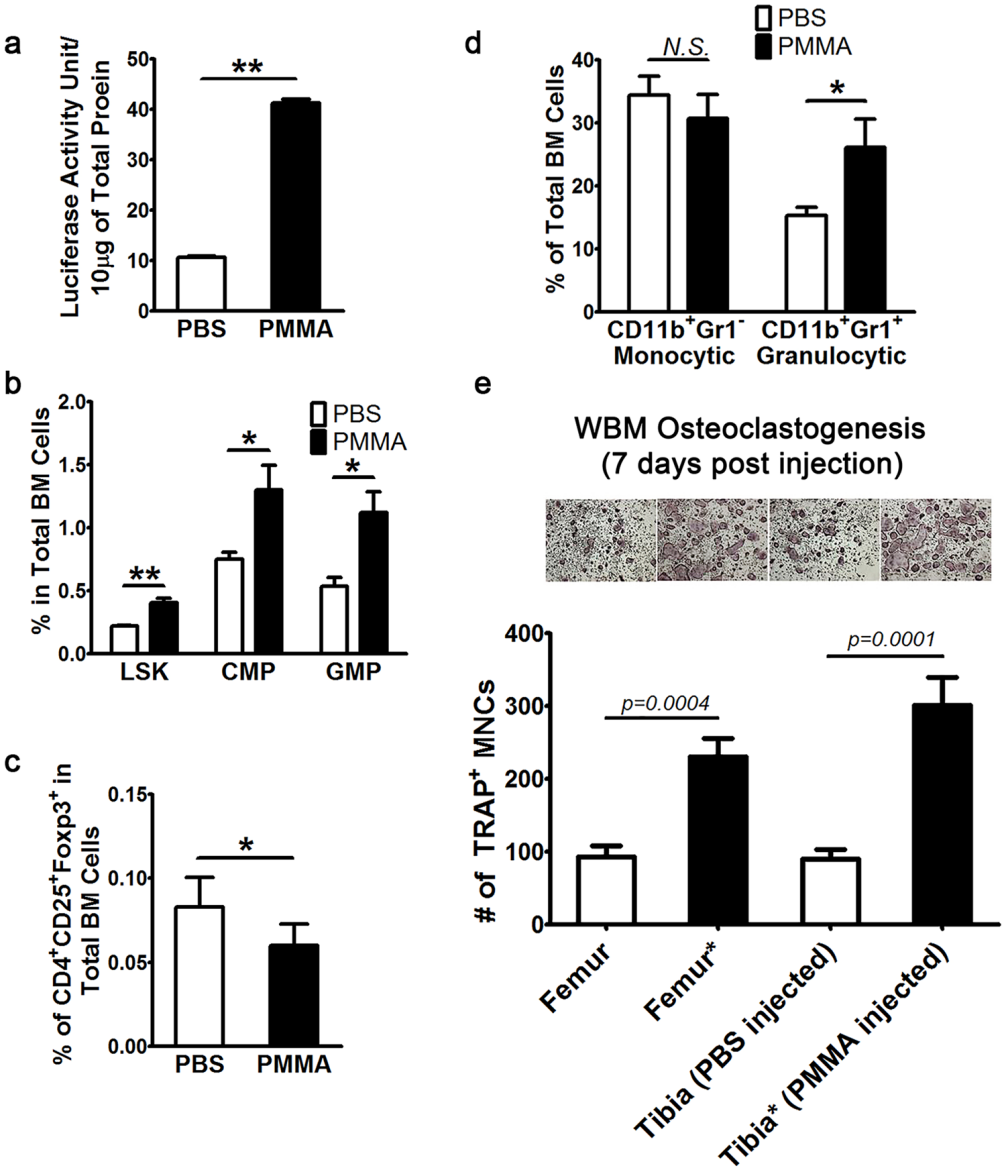
PMMA particles induce NF-κB activity and alter bone marrow cellularity toward reduced immunosuppression. (**a**) PMMA particles induced local NF-κB activation in immune cells. Total cells were isolated from the bone marrow of RelA-Luciferase reporter mice post PMMA intra-tibial injection. After removal of red blood cells, mononucleated cells from bone marrow of separated femurs and tibias were lysed to assess luciferase activity, which was normalized by protein concentration determined by standard BCA assay. (**b**) Increase of myeloid progenitor cells in the bone marrow of mice 2 days post intra-tibial injection of PMMA particles revealed by flow cytometric analysis. 1 × 10^7^ mononucleated bone marrow cells were stained with FACS antibodies to assess myeloid progenitor populations including LSK HSCs, CMPs and GMPs. (**c**) Decreased frequency of bone marrow CD4^+^CD25^+^Foxp3^+^ T_REG_ by intra-tibial PMMA injection. (**d**) Alterations of bone marrow myeloid lineages. Monocytic myeloid cells are marked as CD11b^+^Gr-1^−^ and granulocytic cells as CD11b^+^Gr-1^+^. (**e**,**f**) Assessment of osteoclastogenic potential of whole bone marrow cells (WBM) by *ex vivo* osteoclastogenesis assay (OCgenesis) 2 days after intra-tibial injection of PMMA. 50,000, 100,000 or 200,000 total bone marrow cells were cultured in 96-well plates supplemented with CMG and RANKL to achieve optimal cell density for osteoclast formation. After 4 days of culture, cells were fixed before subjected TRAP staining to visualize osteoclasts. Cells with expanded cytoplasm and more than 3 nuclei were counted as matured osteoclasts. (**e**) Representative images and quantification of the number of multi-nucleated (MNC) osteoclasts per well counted from triplicates of three independent experiments. All columns in the graphs were represented as mean ± SD. *p < 0.05; **p < 0.005 or as indicated by Student T-test.

We have shown previously that PMMA particles robustly activate NF-κB in myeloid cells leading to pro-inflammatory and pro-osteolytic conditions^17,18^. We have also shown recently that activity of NF-κB is exacerbated under conditions of T_REG_ cell inactivity wherein the transcription factor foxp3 is mutated^16^. To further examine the cellular response to PMMA, we established a new experimental mouse model whereby PMMA particles or vehicle were injected directly into the proximal tibia using a 27G syringe. At different time points post injection, *in vivo* NF-κB reporter activity was measured. In addition, myeloid cells and lymphocytes from bone marrow and spleen were FACS sorted and quantified. Our data indicate that NF-κB luciferase activity in the whole bone marrow of PMMA-injected RelA-luc reporter mice was significantly (4 folds) elevated compared with baseline activity in control PBS-injected mice (Fig. 1a). We then used FACS analysis to examine the cellular response locally at the site of PMMA injection, namely the tibia and at adjacent femurs. The data depicted in Fig. 1 indicate that as early as two days post injection, PMMA particles induced two-fold increase in all myeloid progenitor populations examined, including lineage^−^c-Sca-1^+^c-kit^+^ hematopoietic stem cells (LSK HSCs) lineage^−^c-kit^+^CD34^+^Fcγ^−^ common myeloid progenitors (CMPs) and lineage^−^c-kit^+^CD34^+^F4/80^+^ granulocyte-macrophage progenitors (GMPs) (Fig. 1b) while significantly reducing Foxp3^+^ T_REG_ cells in tibia bone marrow (Fig. 1c) and marginally affecting cellularity in adjacent femurs (data not shown). We also examined the compartment of more committed and mature myeloid populations in the bone marrow and found frequencies of CD11b^+^Gr1^+^ granulocytic cells were elevated in PMMA injected tibias, while the frequency of CD11b^+^Gr1^−^ monocytic cells was unaffected (Fig. 1d). Furthermore, these cellular changes summoned a moderately yet significantly elevated osteoclastogenic potential of whole bone marrow cells, indicating PMMA also increased osteoclast progenitors and/or their osteoclastogenic potential at the injection site (Fig. 1e,f).

### PMMA particles modulate extra-medullary hematopoiesis in the spleen

**Figure 2:**
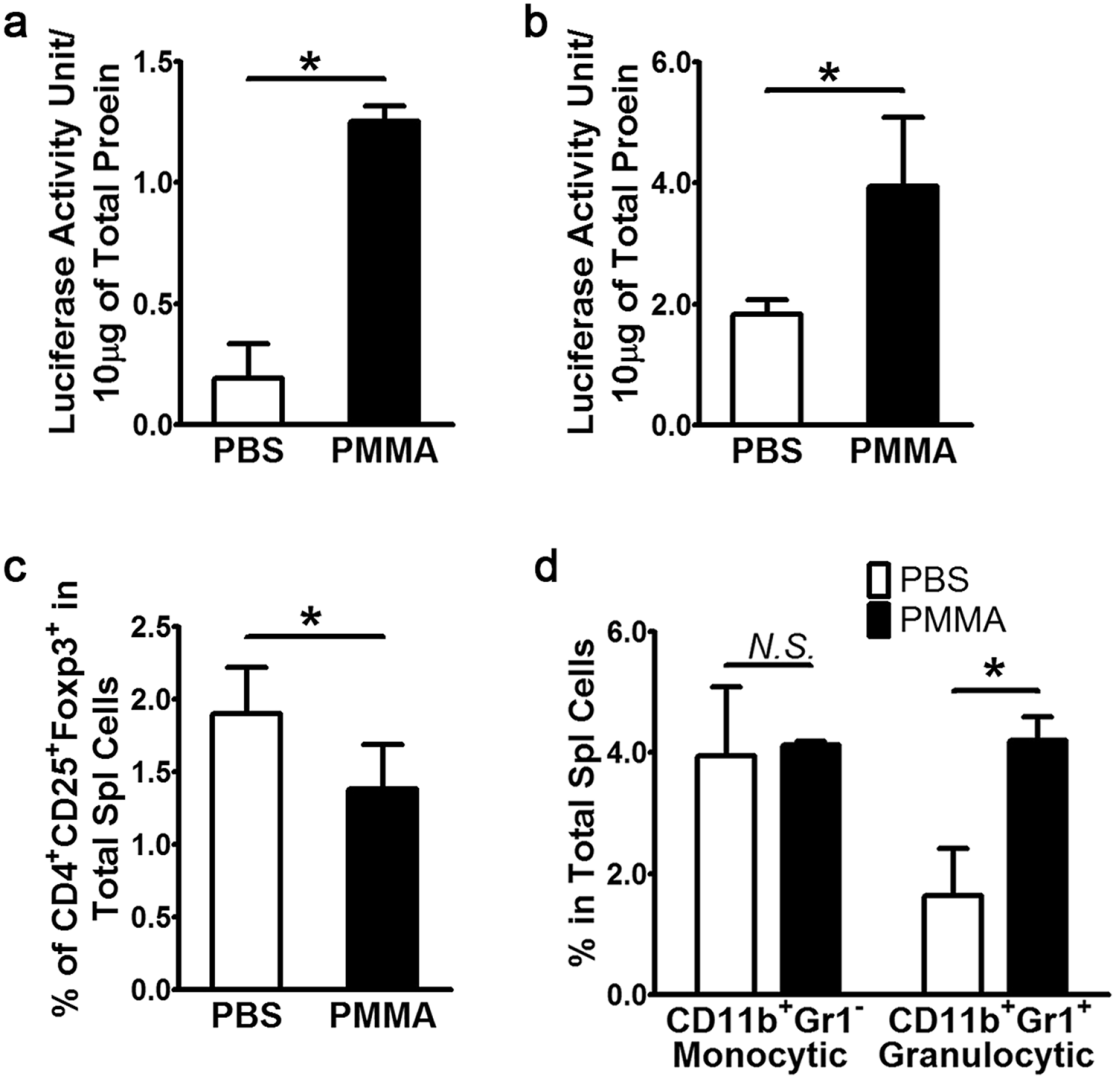
PMMA particles induce NF-κB activity and modulate extra-medullary hematopoiesis in the spleen. (**a**) PMMA particles induced NF-κB activation in immune cells. Total cells were isolated from the spleen from post PMMA intratibially injected animals. After removal of red blood cells, mononucleated cells were lysed to assess luciferase activity, which was normalized by protein concentration determined by standard BCA assay. (**b**) CD4^+^ T helper were further fractionated before measurements of luciferase activity normalized either by total protein input or cell number (not shown). (**c**) Decreased frequency of bone marrow CD4^+^CD25^+^Foxp3^+^T_REG_ in the spleen of PMMA injected animals. (**d**) Alterations of myeloid lineages. All columns in the graphs were represented as mean ± SD. *p < 0.05 by Student T-test.

To evaluate plausible systemic response to PMMA injection in the tibia, we examined NF-κB activity and hematopoiesis in the spleen. Similar to what was observed in the tibia (i.e. local response), NF-κB luciferase activity was also significantly elevated in either whole spleen cells or splenic CD4^+^ T cells (Fig. 2a,b). The number of spleen CD4^+^CD25^+^Foxp3^+^ T_REG_ cells two days post-injection was also significantly reduced (Fig. 2c) whereas frequency of CD11b^+^Gr1^+^ cells that include but not limited to neutrophils and myeloid derived suppressive cells (MDSCs) was significantly increased (Fig. 2d). CD4^+^CD25^+^Foxp3^+^ T_REG_ in the periphery including the blood and lymph nodes were also significantly decreased (Fig. S1). These findings suggest that PMMA particles elicit an acute inflammatory response that extends from pro-inflammatory marrow macrophages to systemic pro-inflammatory neutrophils, granulocytes and immunosuppressive MDSC cells. This further suggests that PMMA-induced changes in the marrow elicit systemic responses, likely cytokine-mediated signaling, to modulate immune responses in the spleen, lymph nodes and peripheral blood. Thus, this inflammatory cascade may initiate a vicious cycle that magnifies the severity of PMMA-induced osteolytic disease.

### The increase of myeloid progenitors in response to PMMA is transient, while the reduction of regulatory T cells is prolonged

**Figure 3:**
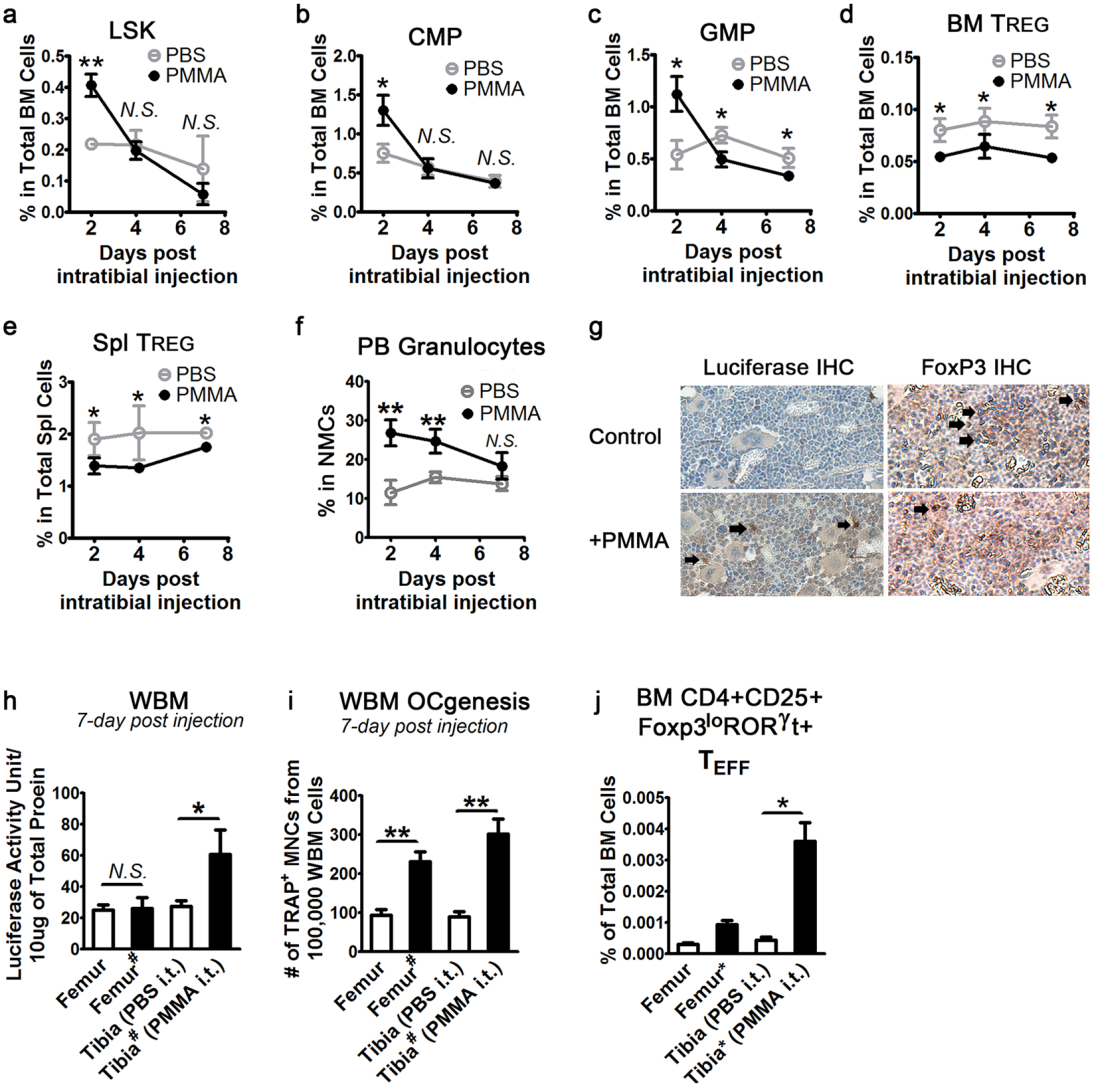
Increase of myeloid progenitors in response to PMMA is transient, while reduction of regulatory T cells is prolonged. (**a**) Frequency of bone marrow myeloid progenitor LSK, (**b**) CMP and (**c**) GMP 2, 4 and 7 days after intra-tibial injection of PMMA. (**d**) Frequency of T_REG_ in the bone marrow (BM) and (**e**) in the spleen (Spl) over time. (**f**) Frequency of granulocytes in the mononuclear peripheral blood cells (PB/MNCs) over time. (**g**) IHC for Luciferase and FoxP3 from Control and PMMA-treated conditions. Arrows point to reactive staining (**h**) Quantification of normalized NF-κB reporter activity in WBM cells isolated from femur and tibia 7 days after intra-tibial (i.t.) injection of PMMA. # denotes femur adjacent to injected tibia. (**i**) Assessment of osteoclastogenic potential of WBM cells by *ex vivo* osteoclastogenesis assay (OCgenesis) 7 days after intra-tibial injection of PMMA. (**j**) % of CD4^+^CD25^+^ FoxP3^lo^RORγt^+^ T cells in the bone marrow (BM) of mice treated as described in H-I. All columns in the graphs were represented as mean ± SD. *p < 0.05; **p < 0.005 by Student T-test.

To further dissect the *in vivo* response of PMMA, we followed the changes of myeloid progenitor and regulatory T cell population in the bone marrow over time from 2 to 7 days after intra-tibial injection of PMMA. To monitor the systemic response, we extended our analyses to cellular changes in the spleen, lymph nodes and blood. We found that the significant increase of myeloid progenitor populations including LSKs, CMPs and GMPs rapidly diminished as early as 4 days post injection (Fig. 3a–c). In contrast, PMMA-induced decrease of T_REG_ frequency remained persistent in the bone marrow and in the spleen of intra-tibially injected animals even after 7 days (Fig. 3d,e). Concomitantly, peripheral blood granulocytic neutrophils were also increased early after injection (2 days) and gradually declined back to the level similar to PBS injected animals (Fig. 3f). Finally, RelA-luciferase reporter activity, a measure of NF-κB activity, was also normalized systemically 7 days after injection, as no significant difference was found between PMMA- and PBS-injected mice in both the spleen and lymph nodes (Fig. S2a,b). However, immunostaining for Luciferase in bone sections from PMMA-injected tibias and luciferase activity of the bone marrow cells derived from PMMA injected tibias remained significantly increased (Fig. 3g,h). Consistent with the observed normalized systemic response, NF-κB activity also declined in femurs one-week post PMMA injection to adjacent tibias (Fig. 3h, marked with #). More importantly, when we performed functional assessment of osteoclast precursor numbers by *ex vivo* osteoclastogenesis assay, whole bone marrow cells from PMMA injected tibia and from adjacent femurs retained significantly higher osteoclastogenic potential than those from the PBS-injected controls (Fig. 3i). Since the numbers of myeloid progenitors were normal at this stage post PMMA injection, PMMA was most likely to potentiate and sensitize osteoclast progenitors versus increase their numbers at early stage. These results suggest that PMMA elicits a prolonged dampening of immunosuppression by T_REGS_, which may lead to unrestrained inflammatory response already triggered by significant increase in frequency of innate immune cells, e.g. macrophages. Although tibial injection resulted in temporal systemic inflammatory response, elevated NF-κB activity persisted at the injection site.

### PMMA particles enhance expression of markers of pathogenic TH effector cells

The increased local and systemic inflammatory burden along with significant reduction in T_REG_ frequency in response to PMMA exposure prompted us to further interrogate changes in T cell populations that may contribute to this pathology. In this regard, it has been shown previously that under inflammatory conditions, T_REG_ cells may lose their immunosuppressive phenotype and assume a T_H_ effector pathogenic phenotype^19–23^. This phenotypic switch depends on suppression or inactivation of Foxp3.

**Figure 4:**
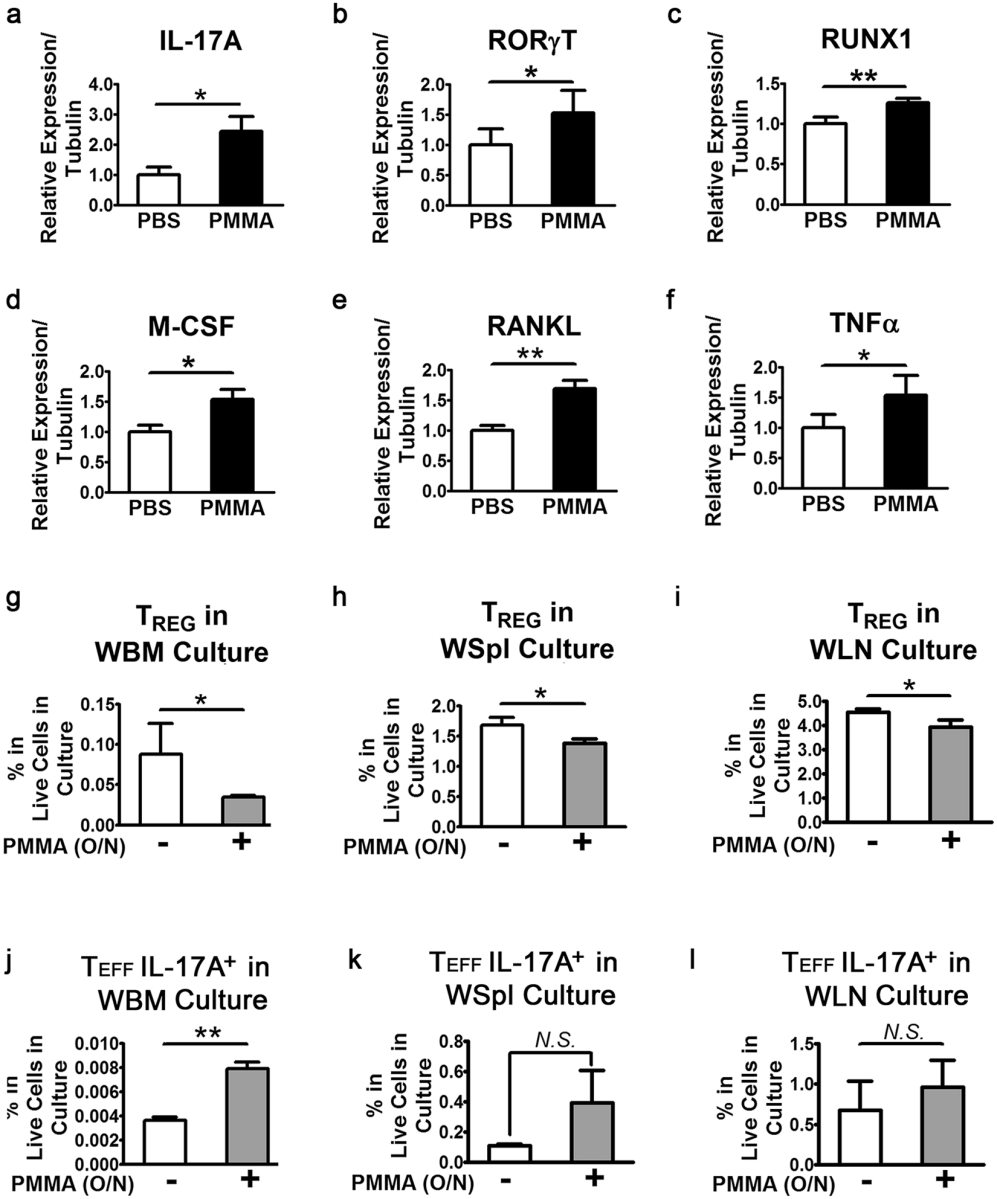
Increase of T_H_17 immunity, upregulation of inflammatory factors produced by effector T cells and recapitulation of the *in vivo* PMMA effect on T_REG_ by *ex vivo* cultures. (**a**–**c**) CD4^+^CD25^−^ effector T cells (T_EFF_) were enriched by magnetic bead sorting (MACS) and lysed in Trizol reagent for RNA isolation. After cDNA synthesis through reverse transcription (RT), quantitative polymerase chain reaction (qPCR) was performed to assess mRNA expression of T_H_17 markers including IL-17A, RORγt and RUNX1. (**d**–**f**) Expression of proinflammatory/pro-osteoclastogenic cytokines including M-CSF, RANKL and TNFα were also measured by qRT-PCR. Expression of tubulin was used as loading control. ΔCq values were calculated by Cq value of each given gene divided by that of tubulin. Plots were generated by values that were normalized with mock PBS injected control group. (**g**–**i**) Frequencies of CD4^+^CD25^+^Foxp3^+^T_REG_ in whole bone marrow (WBM), whole spleen (WSpl) and while lymph node (WLN) cells were assessed by flow cytometry after overnight incubation with 0.4% PMMA. (**j**–**l**) Frequencies CD4^+^CD25^+^CD44^+^IL-17A^+^ T_EFF_ were also analyzed. All columns in the graphs were represented as mean ± SD. *p < 0.05; **p < 0.005 by Student T-test.

Supporting a potential pathogenic switch of T_REG_ cells into T_H_ effector cells, we found that 2 days post intra-tibial injection of PMMA, bone marrow derived CD4+ T effector cells possessed significantly higher percentage of Foxp3^lo^ RORγT^+^ cells (Fig. 3j). Interestingly, utilizing Foxp3 GFP reporter mice in which expression of GFP and Foxp3 are coupled, not only did we observe reduced number of CD4^+^CD25^+^T_REG_ *in situ* (bone marrow), extramedullary (spleen) and in periphery (blood and lymph node) upon intra-tibial injection of PMMA (Fig. S3a–d), it was also accompanied by increased mRNA expression of T_H_17 markers IL-17A, RORγt, RUNX1 and a large number of other inflammatory and osteoclastogenic factors including TNFα, RANKL and M-CSF (Fig. 4a–f). Similar results were also obtained from spleen T_EFF_ cells post PMMA injection (Fig. S4a–f) particularly for RORγt/RUNX1/IL-17, but to a lesser extent for other effector/pro-inflammatory cytokines. These results show increased frequency of T_EFF_ at the expense of T_REG_ cells, suggesting potential T cell phenotype switching.

### *In vivo* effect of PMMA on Treg can be recapitulated *ex vivo*

It is reasonable to postulate that the negative impact of PMMA on T_REGS_ is mediated by secondary mechanisms as it mostly requires the engagement of T-cell receptor (TCR) signaling and antigen presentation by innate immune cells such as dendritic cells and macrophages^24^. However, mechano-transduction of T cells has been recently conceptualized and investigated^25^ and therefore, PMMA may also target T_REG_ (and T_EFF_) in a direct manner. To gain further insights into the effect of PMMA on T cells including both T_REG_s and T_EFF_ (direct vs. indirect mechanism), we conducted *ex vivo* cultures of whole bone marrow (WBM), whole spleen (WSpl) and whole lymph node (WLN) cells. Freshly isolated cells were cultured overnight in the presence of PMMA. These heterogeneous cultures were then stimulated with pan stimulator PMA/ionomycin for 5 hrs. Non-PMMA treated and -PMA/ionomycin stimulated cultured served as controls. Interestingly, in cultures from all 3 sources, percentage of CD3^+^CD4^+^CD25+ T_REG_ was reduced by overnight treatment of PMMA, regardless of PMA/ionomycin stimulation (Fig. 4g–i). Reduction of T_REGS_ was significantly greater in the WBM than in the WSpl and WLN cultures. Furthermore, percentage of IL-17A expressing cells in the CD3^+^CD4^+^CD25^−^ T_EFF_ population was significantly increased in WBM culture by PMMA treatment (Fig. 4j). WSpl derived T_EFF_ cells also exhibited a trend of increased frequency of IL-17A^+^ population in the presence of PMMA (Fig. 4k), while the percentage of IL-17A^+^ T effector cells in the WLN culture was unaltered (Fig. 4l). Because WBMs and WSpls constitute significant proportion of myeloid cells (of the innate immune system; >40% for WBM and >5% for WSpl) compared to WLNs that is considered negligible proportion of myeloid cells (<1%), these data strongly suggest that secondary mechanisms (i.e. myeloid/T-cell interaction) most likely play a larger role in the effect of PMMA on T_REG_ and T_EFF_ cells.

### PMMA particles impair the osteoclast suppressive function of TREGS

**Figure 5:**
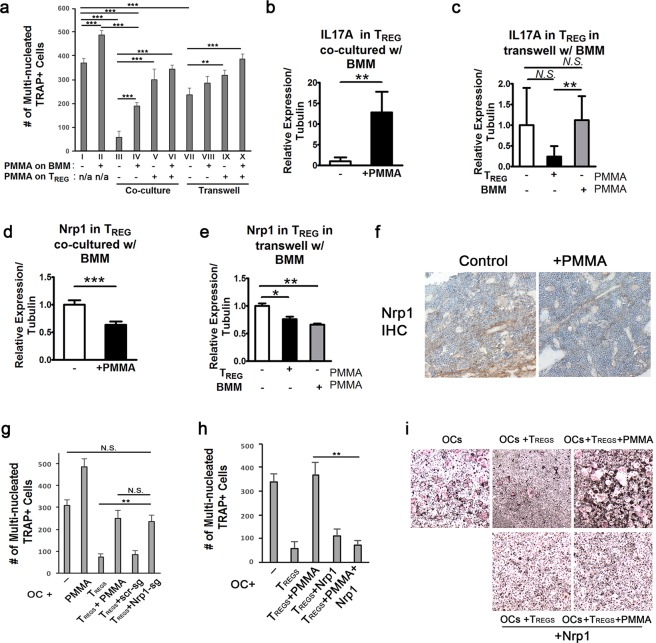
Inhibition of osteoclastogenesis by T_REG_ in the presence of PMMA is Nrp1-mediated event. (**a**) MACS-purified T_REG_ were either co-cultured with bone marrow derived macrophages (BMMs) or placed in the upper chambers of transwell cultures with BMMs in the lower chambers. RANKL and M-CSF were added at the beginning of cell culture and again after 2 days to promote osteoclastogenesis. PMMA particles were added to BMMs or T_REGS_ as indicated. After 4 days, T_REG_ cells were separated from cultures before TRAP staining was performed to visualize osteoclast formation. Results from BMM-T_REG_ co-cultures and BMM-T_REG_ transwell cultures are marked accordingly. (**b**–**e**) Isolated T_REG_ from co-cultures and transwell upper chambers were lysed in Trizol reagent for RNA isolation. cDNAs were subsequently generated for qPCR analysis. (**f**) Immunostaining for Nrp1 in bone sections from control and PMMA-treated mice. (**g**) Osteoclast differentiation (+/−PMMA treated T_REGS_) as described in panel A in the absence or presence of intact T_REGS_ or T_REGS_ transduced with scrambled (scr) shRNA or Nrp-1 shRNA. (**h**) Osteoclast differentiation (as described above) in the absence or presence of vehicle or PMMA-treated T_REGS_. Some conditions include overexpression of retroviral Nrp-1 in T_REGS_ (as indicated). (**i**) Representative images of osteoclast cultures quantified in panel h. All columns in the graphs were represented as mean ± SD. *p < 0.05; **p < 0.005; ***p < 0.001 by Student T-test.

To explore the potential mechanisms by which PMMA affects T_REG_ cell phenotype, we conducted co-culture and transwell studies (Fig. 5). CD4^+^CD25^+^ T_REG_ cells were isolated by MACS and either co-cultured with BMMs or cultured in transwells with BMMs at the bottom of the plate. RANKL was added to BMMs to promote osteoclastogenesis followed by stimulating some wells with PMMA particles. Half of T_REGS_ were treated with PMMA particles and the other half were left untreated. Additionally, 2,000U of human recombinant IL-2 was also supplemented to support immune suppressive function of all CD4^+^CD25^+^ T_REG_ cells. As expected, adding T_REG_ cells to BMMs in co-culture drastically inhibited RANKL stimulated osteoclastogenesis (Figs 5a and S5; compare corresponding panels I, II and III in both figures). Interestingly, whereas T_REGs_ in co-culture with BMMs sufficiently interfered with PMMA-exacerbated osteoclastogenesis (Figs 5a and S5; corresponding panel IV), pre-treatment of T_REGS_ with PMMA particles prior to co-culture with BMMs impaired T_REGS_ anti-osteoclastogeneic function (Figs 5a and S5, panels V and VI compared with panel III). This impaired T_REG_ function was detected in both co-culture and transwell (Fig. 5a, panels VIII and IX) conditions. Notably, when PMMA was added to the BMM compartment, T_REGS_ were only partially able to inhibit the exacerbated osteoclastogenesis (Fig. 5a; panels IV and VIII). Interestingly, when we monitored mRNA expression of pro-inflammatory cytokines to assess reprogramming of T_REGS_, we observed a 12-fold increase of IL-17A expression in the T_REG_ co-cultured with BMM stimulated with PMMA (Fig. 5b). On the other hand, we did not see significant changes of IL-17A expression or other inflammatory cytokines in the T_REG_ cultured in transwells with BMMs undergoing PMMA exacerbated osteoclastogenesis (Fig. 5c and data not shown). These observations suggest that T_REG_ cells, while partially affected by direct contact with PMMA, they require direct cell- cell contact with BMMs to achieve potent anti-osteoclastogenic potential. The data also suggest that T_REGS_ secreted factors appear to play a role in their anti-osteoclastogenic function, albeit to a lesser degree than direct cell-cell contact. Most importantly, the data suggest that PMMA particles, through their action on T_REGS_, alter the phenotype of these cells from suppressors to IL-17A expressing T cells, suggesting a phenotypic switch.

### PMMA particles impede the T_REG_ anti-osteoclastogenic function by reprogramming T_REGS_ through inhibition of neuropilin-1

To further delineate the mechanism affected by direct impact on T cells, qRT-PCR analysis was performed to survey mRNA expressions of genes that are important for the immune suppressive function of T_REG_ and/or stability of T_REG_. To this end, we observed slight upregulation of Icos and GITR, and no change for Eos and CTLA4 in the presence of PMMA (Fig. S6a–f). We also did not see any changes of CTLA4 expression in T_REG_ cells among all culture conditions (Fig. S5g,h). However, among the markers tested, only neuropillin-1 (Nrp-1) was downregulated in the T_REG_ cells under all culture conditions with PMMA (Fig. 5d–f). Most notably, loss of Nrp-1 expression (resulting from exposure to PMMA) or by shRNA (Figs 5d and S5) rendered T_REGS_ incapable of inhibiting osteoclastogenesis (Figs 5g and S5). Most interestingly, retroviral expression of Nrp-1 in T_REGS_ halted the negative effect of PMMA on these cells and strongly stabilized and augmented their anti-osteoclastogenic function (Fig. 5h,i). These observations suggest that stable expression of Nrp-1 and its downstream signaling, stabilizes T_REGS_ and supports its immunosuppressive function. The data further suggest that danger signals, such as PMMA particles, cause diminution of Nrp-1, which triggers T_REG_ to undergo phenotypic switch to T_EFF_ (i.e. exT_REG_), and exacerbate osteoclastogenesis.

## Discussion

In this study we show that intra-tibial injection of PMMA particles increases the frequency of premature myeloid progenitors LSKs, granulocytic CD11b^+^Gr1^+^ cells and reduce frequency of CD4^+^CD25^+^ T_REG_ cells. We further show that NF-κB reporter activity is significantly increased *in vivo* concurrent with inhibition of Foxp3 and elevated expression of T_H_17 transcription factors Runx1 and RORγt, leading to over production of inflammatory and osteoclastogenic factors such as TNFα, RANKL, M-CSF and IL-17A. These observations suggest that PMMA particles specifically attenuate T_REG_ suppressive activity by inhibiting Foxp3 and switching the T cell phenotype from immunosuppressive to pathogenic.

In previous work, we have shown that PMMA particles exacerbate osteoclastogenesis in whole bone marrow cultures. Thus, it is reasonable to suggest that PMMA particles elicit an inflammatory microenvironment that alters hematopoiesis, highlighted by increased frequency of primitive progenitors such as LSKs. Concurrently PMMA particles induce conditions favorable for suppressing T_REGS_ and promoting pathogenic T_H_17 cell through down regulation of Foxp3. Taken together, the sum of these changes lead to higher inflammatory and osteoclastogenic burdens. Indeed, Foxp3 is indispensable for T_REG_ suppressive function. Mechanistically, Bettelli *et al*.^26^ found that Foxp3 associates with NFAT and NF-κB proteins and hinders their transcriptional activity. In fact, we and others have shown that myeloid and T cells derived from scurfy mice, which harbor inactive mutant Foxp3, exhibit high levels of NFAT and NF-κB activity, supporting the notion that Foxp3-expressing T_REG_ cells suppress effector T helper cells^16^. In this study, using foxp3-GFP reporter mice, we provide direct evidence supporting T_REG_ phenotype switch in response to the PMMA inflammatory signals. Specifically, we observe reduced number of CD4^+^CD25^+^ T_REG_ *in situ* (bone marrow), extramedullary (spleen) and in periphery (blood and lymph node) upon intra-tibial injection of PMMA, concurrent with increased mRNA expression of T_H_17 markers RORγt, and IL-17A. Consistent with the paradigm that Foxp3 provides a transcriptional switch in T cell differentiation, it has been shown that T helper cells, specifically T_H_17, may originate from Foxp3^+^ T_REG_ cells^20^. According to this study, inflammatory conditions render Foxp3 unstable leading to trans-differentiation of T_REG_ cells that just lost Foxp3 expression, so-called exFoxp3, into T_H_17 pathogenic cells. It was further shown that this cell phenotype conversion was mediated by IL-6 and was associated with increased expression of IL-23R, RANKL, and Chemokine Receptor 6 (CCR6). More importantly, these exFoxp3 T_H_17 pathogenic cells were primarily located at sites on inflammation in arthritic joints. Our data suggest that PMMA particles destabilize T_REGS_ through diminution of Foxp3.

Previous studies have shown that inflammatory conditions, such as synovial joint inflammation, destabilized foxp3 in T_REGS_ leading to impaired cell function^27^, increased T_H_17 effector cells and unleashed the activity of the proinflammatory transcription factors NFAT and NF-κB. Multiple mechanisms are involved in the control of T_REG_ stability^28^. These include PI3 kinase/Foxo^29^, Nrp-1/semaphorin-4a/Foxo3a^30^, GITR signaling^31^ and USP21 deubiquitinase^32^. Moreover, several other genes also participate in the balance between T_REG_ and T_H_17 cells such as Foxo3a/TSC1^33,34^, TCR and cytokine signaling integrated by Itk^35^, Tpl2^36^, Eos^37^, TNF/TNFR2^38^, Notch^39^, cooperated signaling between TCR and CD28/CTLA4^40^. Interestingly, when we survey expression of these genes to determine the potential targets of PMMA-mediated T_REG_ instability, we found that Nrp-1 expression was significantly downregulated. Thus, the mechanism underlying T_REG_ reprogramming (e.g. reduced foxp3 expression) appears to involve down regulation of Nrp-1 via undefined stress or danger-like apparatus. As a result, NF-κB activity is elevated leading to induction of pro-inflammatory and osteoclastogenic factors including TNF, IL-17A, RANKL and M-CSF. This ultimately leads to increased osteoclastogenic burden through increased myeloid progenitor numbers and increased CD11b^+^Gr1^+^ myeloid cells. Furthermore, since Nrp-1 is a cell-surface protein/receptor, it is reasonable to speculate that PMMA particles interact with Nrp-1 and elicit mechano-transduction signals. In fact, our data indicate that primed spleen CD4^+^CD25^+^ T_REG_ (stimulation with anti-CD3/CD28 beads for 72 hours) exhibited decreased Foxp3 protein expression in the presence of PMMA. Moreover, CD4^+^CD25^−^ T_EFF_ culture under the same conditions with much lower concentration of supplemented IL-2 (30U rather than 2,000) possessed higher RORγt^+^ population. Perhaps the most compelling evidence supporting this phenomenon is our finding that restoring expression of Nrp-1 via viral transduction stabilized the suppressive phenotype of T_REGS_ and render these cells irresponsive to the inflammatory impact of PMMA particles. These data suggest that PMMA, beyond its conventional role in innate immunity (targeting macrophages; the precursors of osteoclasts), could directly impact the adaptive immunity with unknown mechanisms that require further investigation.

It is well documented that the CTLA4 is required for inhibition of osteoclastogenesis by T_REG_ through direct cell- cell contact, at least *in vitro*. This T_REG_-osteoclast interaction is mediated by CTLA4 expressed by T_REG_ and CD80/86 expressed by osteoclast. Not only CD80/86 osteoclast can escape inhibition by T_REG_, more convincingly CD80/86 deficient mice are osteopenic and exhibit increased osteoclast differentiation *ex vivo*. That said, we did not see any change in mRNA expression of CTLA4 in T_REG_ co-cultured with BMM undergoing PMMA-exacerbated osteoclastogenesis. Ultimately, functional tests will be required with crucial reagents such as CTLA4-Fc to rule out whether PMMA can affect CTLA4-mediated (or GITR, PD-1 etc.) osteoclastogenesis suppression by T_REG_. It is important to note that murine CD4^+^CD8^+^FoxP3^+^ T_REGS_ inhibit osteoclastogenesis in a cell contact-dependent manner with minor contribution by circulating anti-inflammatory cytokines^41^. In contrast, human FoxP3^+^T_REGS_ induce alternative activation and inhibit osteoclast differentiation from peripheral blood monocytes independent of cell contact, yet in a TGFβ and IL-4-dependnet manner^42,43^. Although varying experimental conditions may contribute to this discrepancy, it would be of interest to simultaneously determine Nrp1 expression and function in these two systems.

Our co-culture experiments show that while T_REGS_ potently inhibit osteoclastogenesis, exposure of T_REGS_ to PMMA particles impair their anti-osteoclastogeneic function. Moreover, under co-culture conditions, PMMA particles induce robust expression of proinflammatory cytokines by T_REG_ cells, suggesting that macrophage-T_REG_ contact is crucial for this response. This observation, which is highlighted by secretion of IL-17A and TNFα by exT_REG_ cells, is reminiscent of T_REG_ phenotype switching and assuming an effector function. On the other hand, marginal osteoclast inhibition by T_REGS_ in transwell conditions, suggest that PMMA particles may marginally induce secretion of soluble factors by macrophages that adversely influence secretion of repressors factors by T_REG_ cells. These observations are consistent with the established paradigm wherein T_H_17 serve as osteoclastogenic T_H_ cell type linking T cell activation with bone resorption through the interleukin IL23-IL17 axis^44^. Further delineation of the subsets of pathological effector T cells induced by orthopedic particles vs. rheumatoid diseases may have significant therapeutic implications.

In sum, our findings show that, in addition to its well documented direct effect on osteoclasts, PMMA particles also induce inflammatory osteolysis by modulating T_REG_ cells. Specifically, we suggest that PMMA particles, by yet to be defined mechanism, down regulate Nrp-1 leading to reduced foxp3 and subsequent reprogramming T_REGS_ into T_H_17 pathogenic cells. These cells express pro-inflammatory and osteoclastogenic factors that directly expand the osteoclast progenitor population and exacerbate osteoclastogenesis. Our findings identify the Nrp-1 pathway as a potential therapeutic target to combat inflammatory osteolysis.

## Materials and Methods

### Study design and statistical analysis

Data is expressed as mean ± SD of at least three independent experiments. Typically, each experimental design includes triplicates of each condition. *p < 0.05; **p < 0.005 using Student *t*-test. Our experimental design is based on reaching 0.05 significance and effect size of 25%. With desired difference of 80%, we calculated sample size as 6 mice per group. Experiments were conducted with male and female mice at equal proportions. There are no reported osteolytic differences between mouse sexes.

### Mice

Approval for using animals was obtained from Washington University School of Medicine Institutional Animal Care and Use Committee in accordance with NIH guidelines prior to performing this study. Mice were housed at the Washington University School of Medicine barrier facility. NF-κB reporter (NGL) mice were purchased from Jackson Laboratories to monitor *in vivo* NF-κB activity longitudinally during disease progression as well as *ex vivo* studies including cultures of macrophages/monocytes, proinflammatory T helper cells and co-cultures. Foxp3-GFP reporter mice were kindly provided by Dr. John DiPersio’s lab (Washington University) and were originally from Jackson Laboratories (Bar Harbor, ME USA). The Foxp3 reporter mice were used to perform hematological diagnosis by flow cytometry for T_REG_ cells, myeloid populations and progenitors after manipulation.

### Chemicals and reagents

PMMA particles (Polyscience) were sterilized before injection by washing with 70% EtOH for three times followed by 3 times in PBS and finally resuspended in PBS (0.2 mg/ml for *in vitro* studies and 20 ul per injection of 5 mg/ml *in vivo*). All FACS antibodies, buffers and reagents were purchased from either BD Biosciences, eBioScience/Thermo Fisher or BioLegend.

### Flow cytometry

To analyze myeloid progenitor in the bone marrow, freshly flushed WBM cells were sequentially stained with PE conjugated anti-CD34 and Brilliant Blue 421 conjugated anti-CD16/32 antibodies for 30 minutes on ice, and biotin conjugate lineage antibody cocktail (anti-CD2, -CD3ε -IL7R, -Ter119 and –B220) and PerCP Cy5.5 anti-CD11b, PE Cy7 anti-CD115 (c-fms), Alexa 700 anti-Ly6G and APC H7 anti-CD117 (c-kit) antibodies for additional 30 minutes. After washed with FACS buffer, antibody labeled cells were stained with Brilliant Blue 510 conjugated streptavidin for 20 minutes before analyzed on flow cytometer. To phenotype T cells, whole bone marrow (WBM) or whole spleen (WSpl) cells were stained with PE anti-CD4, PerCP Cy5.5 anti-CD44, APC anti-CD62L, PE Cy7 anti-CD3ε, APC e780 anti-CD8a and Brilliant Blue 421 anti-CD25 for 30 minutes on ice.

### Intra-tibial injection mouse model to test acute/short-term cellular response to PMMA

Mice were anesthetized with 100 μl of ketamine/xylazine cocktail per 10 grams of body weight. Skin above the knee cap was wetted with 70% EtOH to sterilize and visualize the injection site. To allocate the growth plate of tibia, patellar ligament was used as a landmark. 27G needle was inserted above the patellar ligament until encountering resistance. Drill motion was applied to the needle until the growth plate was penetrated. 20 μl of PBS or PMMA solution (5 mg/ml) was released into the bone marrow cavity slowly to avoid back flash.

### Cell Isolation and culture

#### Bone marrow macrophages/monocytes (BMMs)

Bone marrow cells were harvested from femurs and tibias. After cell numbers were determined by hemacytometer counts, cells were cultured in DMEM supplemented with 10% FBS and 10% CMG that contained M-CSF.

#### CD4^+^CD25^+^T_REG_

CD4^+^CD25^+^ cells were isolated from mouse spleens by MACS according to manufacturer’s protocol. After isolation, purity was checked by FACS before cultured with RPMI 1640 medium supplemented with 10% FBS, sodium pyruvate, non-essential amino acids, glutamine, 10 mM HEPES and 50 μM β-mercaptoethanol. To activate T_REG_ at phtsiological level, Dynabeads were added at bead-to-cell ratio of 2:1 and 2,000 U of recombinant IL-2 was also supplemented.

#### CD4^+^CD25^−^ T effector cells

CD4^+^CD25^−^ cells were obtained during CD4^+^CD25^+^T_REG_ cell isolation. T_EFF_ cell culture was supplemented with 30U IL-1 and anti-CD3/CD28 Dynabeads at bead-to-cell ratio of 1:1 for activation and expansion.

#### Spleen and lymph nodes

To isolate single cells from the spleen or inguinal lymph nodes, tissues were carefully dissected, placed in ice-cold FACS buffer, grinded using the back end of a 3-ml syringe before passing through a sterile 70 micro filter. After red blood cell lysis, mononucleated cells were then subjected to either FACS analysis, MACS isolation for CD4^+^ T cells or T_REGS_, or luciferase activity assay.

#### BMM-T_REG_ co-culture and transwell culture for osteoclastogenesis assay

BMMs were prepared as aforementioned. *In vitro* expanded T_REG_ cells were generated by culturing MACS-isolated splenic naïve CD4^+^ T cells in T_REG_ cell differentiation media (R&D Systems), either with or without the presence of PMMA particles for 2 days before directly added onto BMMs for co-culture or into transwell inserts on top of BMMs for transwell culture at BMM-T_REG_ ratio of 5:1 or 10:1.

#### Luciferase assay

Luciferase assay was conducted according to the manufacturer’s protocol (Promega). Briefly, freshly isolated or MACS purified cells were lysed in passive cell lysis buffer After protein concentration was determined by BCA assay, 20 μg of protein was used from each lysate with Luciferase Assay Reagent to measure the light produced by a luminometer.

#### Nrp-1 knockdown

The lentiviral sgRNA vectors and sequences for targeting Nrp-1 were designed by the Genome Center at Washington University. The lentiviral sgRNA bearing a scrambled, non-specific sequence was used as control. To generate lentiviral particles, each vector was co-transfected into HEK cells together with packaging and helper vectors, replenished with fresh media on the next day. Conditioned media containing lentiviral particles was collected after 2 days, concentrated, and stored at −80 upon use. To transduce T cells, 3 million spleen derived mouse naive CD4+ T cells were incubated with 300 ul concentrated lentiviral stock in the presence of 10 ug/ml polybrene overnight, washed and replenished with T_REG_ cell differentiation media, in the presence or absence of PMMA particles for 2 days before proceeding to co-culture experiment with BMMs.

#### Nrp-1 expression (Gain of function)

To achieve expression of Nrp-1 in Tregs, retroviral vector (namely PINCO) carrying mouse wildtype Nrp-1 gene was purchased from Addgene (https://www.addgene.org/browse/gene/18186/). To generate retroviral particles, 5 ug of each PINCO vector plasmid was transfected into 4 millions of PLAT-E cells plated on p100 TC dish 1 day prior. After 8 hours, transfected PLAT-E cells were replenished with RPMI media supplemented with 10% FBS and 50 uM b-ME. Supernatants were then collected after 48 hours of culture and served as retroviral stocks, which were kept on ice until use. To transduce T_REGS_, 1 millions of freshly MACS-isolated naïve CD4+ T cells from mouse spleen were incubated overnight with 3 ml of retroviral stock supplemented with reagents from the T_REG_ Cell Differentiation Kit (R&D Systems) and 10ug/ml of polybrene. Transduced naïve CD4+ T cells were then washed and replenished with T_REG_ differentiation media and cultured for 2 additional days before subjected to co-culture with osteoclast progenitors for *in vitro* osteoclastogenesis assay. To condition PINCO-Nrp1 transduced T_REGS_, 0.1 mg/ml PMMA particles were added to culture.

#### Gene expression analysis by RT-qPCR

Cells isolated by MACS or harvested freshly from cultures were lysed in Trizol reagent. Total RNAs were isolated and cDNA synthesis was performed according to the manufacturers’ protocol. 20 μl of each cDNA sample was diluted by 10-fold with Tris-EDTA buffer and 4 μl of diluted cDNA sample was used for 10 μl qPCR reaction with SYBR Green PCR mix. Primers for assessing cytokine expressions (IL-10, IL-17A, M-CSF, RANKL and TNFα) were described previously (Chen *et al*. 2015). Other primer sequences are listed as follows – For **CTLA4**, forward primer 5′-GCTTCCTAGATTACCCCTTCTGC-3′, reverse primer 5′-CGGGCATGGTTCTGGATCA-3′; for **Dbc1**, forward primer 5′-GTATCTCAGTGCAGCCCTCC-3′, reverse primer 5′-AACGGGCAAACTCCCTGTAT-3′; for **Eos**, forward primer 5′-TCTGGACCACGTCATGTTCAC-3′, reverse primer 5′-ACGATGTGGGAAGAGAACTCATA-3′; for **Foxp3**, forward primer 5′-ATTGAGGGTGGGTGTCAGGA-3′, reverse primer 5′-ACAGCATGGGTCTGTCTTCTC-3′; for **GATA3**, forward primer 5′-CCATTACCACCTATCCGCCC-3′, reverse primer 5′-TTCACACACTCCCTGCCTTC-3′, for GITR, forward primer 5′-CCACTGCCCACTGAGCAATAC, reverse primer 5′-GTAAAACTGCGGTAAGTGAGGG-3′; for **Helios**, forward primer 5′-GAGCCGTGAGGATGAGATCAG-3′; reverse primer 5′-CTCCCTCGCCTTGAAGGTC-3′; for **Icos**, forward primer 5′-ATGAAGCCGTACTTCTGCCG-3′, reverse primer 5′-CGCATTTTTAACTGCTGGACAG-3′; for Nrp-1, forward primer 5′-GACAAATGTGGCGGGACCATA-3′, reverse primer 5′-TGGATTAGCCATTCACACTTCTC-3′; for **RUNX1**, forward primer 5′-CAGGCAGGACGAATCACACT-3′, reverse primer 5′- CTCGTGCTGGCATCTCTCAT-3′; for **ROR**γ**T**, forward primer 5′-TACCCTACTGAGGAGGACAGG, reverse primer 5′-AATGGGGCAGTTCTGCTGAC-3′; for **Tbet**, forward primer 5′-GTCTGGGAAGCTGAGAGTCG-3′, reverse primer 5′-ACATTCGCCGTCCTTGCTTA-3′, for **Tpl2**, forward primer 5′-ATGGAGTACATGAGCACTGGA-3′, reverse primer 5′-GGCTCTTCACTTGCATAAAGGTT-3′; for **Ubc 13**, forward primer 5′-ACAAGAGCAGAGGCCGAAC′3′, reverse primer 5′-GCAAACGCTGGGTTTCCTTG-3′.

#### Immunohistochemistry

At the end of experiments, mouse long bones were harvested and fixed in 10% neutral buffered formalin for 24 hours followed by decalcification in Immunocal (StatLab, McKinney, TX) for 3 days. Tissues were then processed, embedded into paraffin, and sectioned 5 mm thick. For immunohistochemistry, sections were de-paraffinized and rehydrated using xylene followed by ethanol gradient. Antigen retrieval was performed by incubating samples at 60 degrees celsius in Citrate buffer (pH 6.0) followed by quenching of endogenous peroxidase activity with 3% H_2_O_2_. Sections were blocked using DAKO solution with background reducing components. Sections were incubated overnight with a 1:200 dilution of anti-Luciferase (Novus), anti-Nrp1 (Novus) or anti-FoxP3 (Novus) antibody. Sections were rinsed in phosphate-buffered saline (PBS) followed by a 1:1000 dilution of biotinylated secondary antibody for one hour. Post-secondary antibody incubation, the sections were incubated with stereptavidin-HRP (2 ug/ml) for 20 min. After extensive washing with PBS, sections were developed using Impact DAB kit (Vector Biolabs).

## Supplementary information

Supplementary figures
