# Supplementary material for: Inflammatory Responses Reprogram TREGS Through Impairment of Neuropilin-1
Source: Sci Rep. 2019 Jul 18;9:10429. doi: 10.1038/s41598-019-46934-x (PMC6639378; doi:10.1038/s41598-019-46934-x)
Supplement: Supplementary file 1 — Supplementary figures [file 41598_2019_46934_MOESM1_ESM.pdf]

Supplemental Material

Inflammatory Responses Reprogram T_{REGS} Through Impairment of Neuropilin-1

Tim Hung-Po Chen, Manoj Arra, Gabriel Mbalaviele, Gaurav Swarnkar and Yousef-Abu-Amer.

Figure S1.

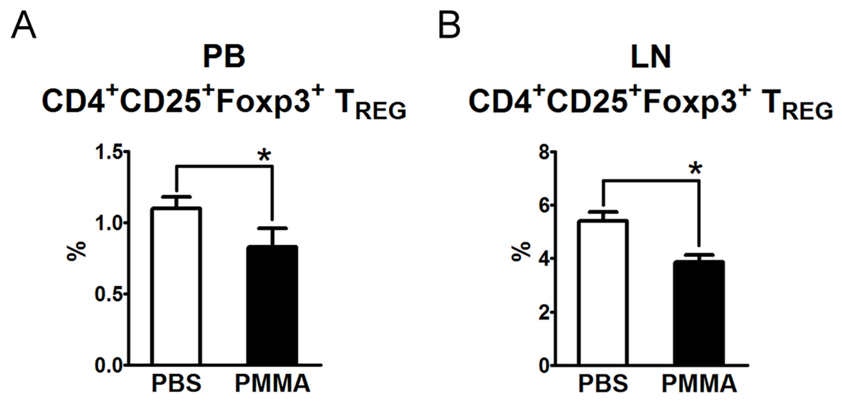

Figure S1. PMMA particles reduced T_{REG} number in (A) peripheral blood (PB) and (B) lymph nodes *in vivo*. Two days after intra-tibial injection of PMMA, peripheral blood samples were obtained from the facial vein, axillary, brachial and inguinal lymph nodes were dissected. Mononucleated cells from the peripheral blood were obtained by removal of red blood cells with red lysis buffer. Mononucleated lymph nodes cells were harvested by mincing tissues with the backend of the plunger of a 3-ml syringe followed by red cell removal. FACS staining of cell surface markers was performed before cells were fixed and permeabilized for nuclear staining of Foxp3. All columns in the graphs were represented as mean±SD. * $p<0.05$ by Student T-test.

Figure S2.

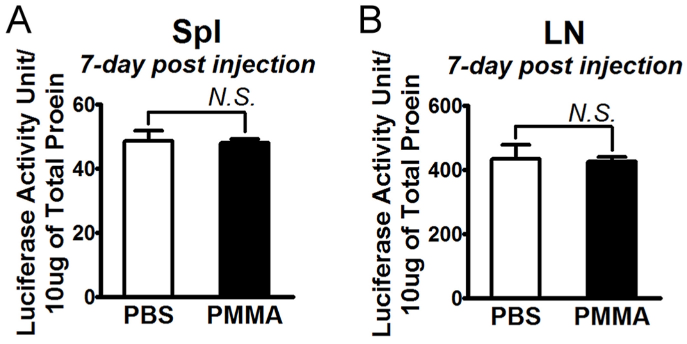

Figure S2. Diminishing systemic NF-κB activation one week after intra-tibial injection of PMMA to NF-κB reporter mice. Mononucleated cells obtained from (A) the spleen (Spl) and lymph nodes (LN) were lysed in passive lysis buffer. After protein concentration was determined, 20 μg of each lysate was used for assessment of luciferase activity. All columns in the graphs were represented as mean±SD by Student T-test.

Figure S3

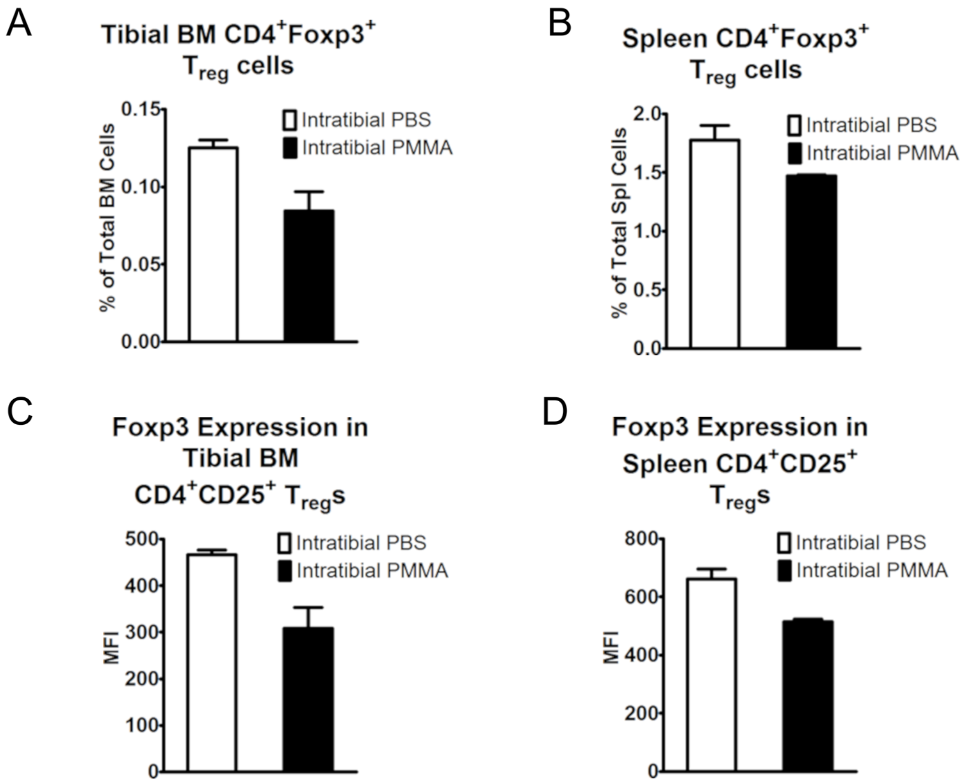

Figure S3. Decrease of Foxp3$^+$ T$_{REG}$ cells, reduced expression of Foxp3 in T$_{REG}$ and conversion of T$_{REG}$ to T$_H$17 could be recapitulated in B6 Foxp3 GFP reporter mice upon intra-tibial injection of PMMA. (A) Frequency of CD4$^+$CD25$^+$Foxp3$^+$ T$_{REG}$ in the tibia. (B) Frequency of CD4$^+$CD25$^+$Foxp3$^+$ T$_{REG}$ in the spleen. (C) Expression of Foxp3 protein in tibial CD4$^+$CD25$^+$ T$_{REG}$ expressed as mean fluorescence in cells (MFI). (D) Expression of Foxp3 protein in splenic CD4$^+$CD25$^+$ T$_{REG}$.

Figure S4

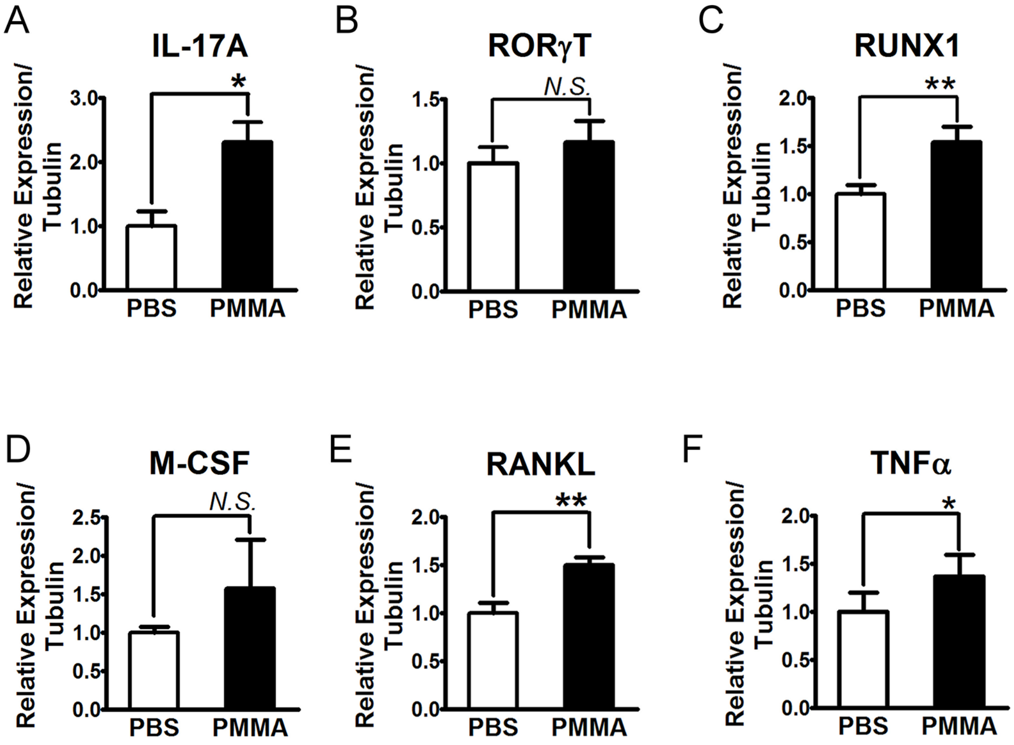

Figure S4. Increase of T_{H17} immunity and production of proinflammatory cytokines in splenic T effector cells post intra-tibial PMMA injection. CD4+CD24^{-} T effector cell were isolated by MACS and lysed in Trizol reagent to obtain total RNA. After cDNA synthesis, expressions of (A) IL-17A, (B) RORγT, (C) RUNX1, (D) M-CSF, (E) RANKL and (F) TNFα were assessed by qPCR. All columns in the graphs were represented as mean±SD. * $p<0.05$; ** $p<0.005$ by Student T-test.

Figure S5

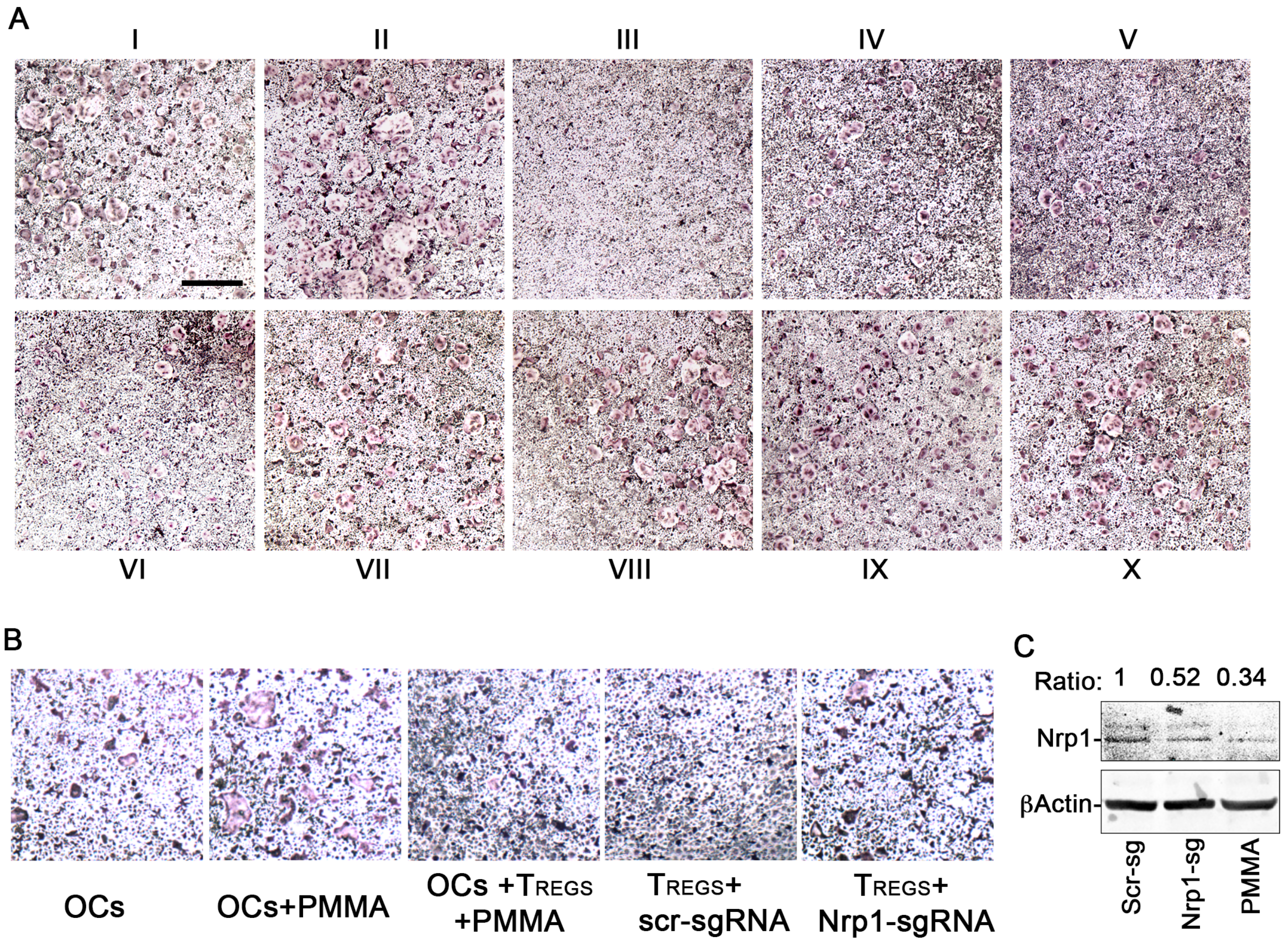

Figure S5. Representative images of osteoclasts supporting Figure 5a. (A) BMMs and T_{REGS} were plated as described in fig 5. Representative images were taken at 4x objective. Bar represents 1µm. (B) representative osteoclast images from selected osteoclast and T_{REG} culture condition (control, PMMA, sgRNA) corresponding to data in fig 5G. (C) Nrp-1 Western blot of T_{REGS} transduced with scrambled (scr) sgRNA, Nrp1-sgRNA, or treated with PMMA for 48 hrs. Nrp-1/β-actin ratio is indicated on top.

Figure S6

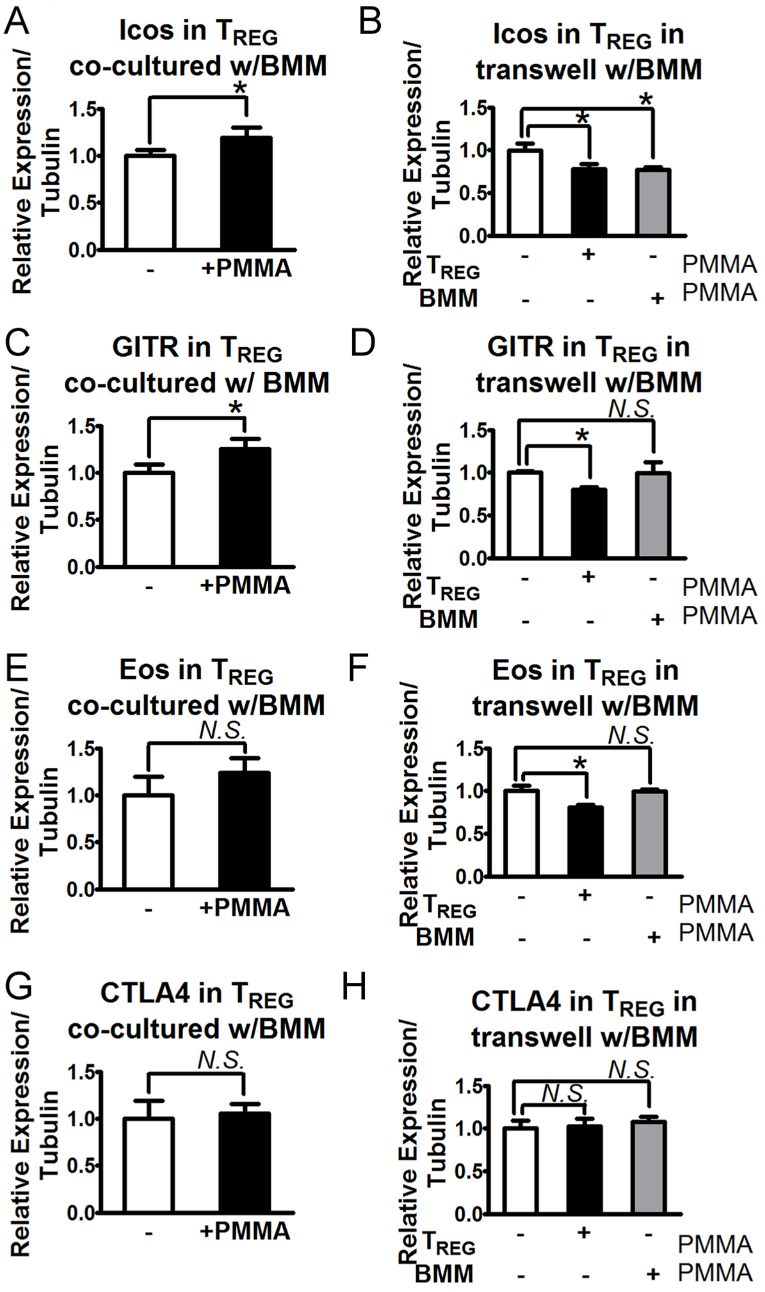

Figure S6. Assessment of PMMA induced instability of T_{REG} cells upon interaction with bone marrow macrophages *in vitro.* (A, C, E, G) $CD4^{+}CD25^{+}$ T_{REG} cells with or without the presence of PMMA were co-cultured with RANKL stimulated bone marrow macrophages (BMMs). After separating from BMMs, these cultured T_{REG} cells were subjected to RNA isolation and subsequent qRT-PCR analysis. (B, D, F, H) $CD4^{+}CD25^{+}$ T_{REG} cells placed in the transwell chamber and co-cultured with RANKL stimulated BMMs were isolated for total RNA isolation and subsequent qRT-PCR analysis.

PMMA was either added to the T_{REG} or the BMM compartment. Expression of the T_{REGS} markers Icos, GITR, Eos and CTLA4 was measured using RT-QPCR. All columns in the graphs were represented as mean±SD. * $p<0.05$ by Student T-test.
